# A retrospective review of vital signs and clinical outcomes of febrile infants younger than 3 months old presenting to the emergency department

**DOI:** 10.1371/journal.pone.0190649

**Published:** 2018-01-05

**Authors:** Shu-Ling Chong, Gene Yong-Kwang Ong, Wendy Yi Wen Chin, John Mingzhou Chua, Praseetha Nair, Alicia Shu Zhen Ong, Kee Chong Ng, Ian Maconochie

**Affiliations:** 1 Department of Emergency Medicine, KK Women’s and Children’s Hospital, Singapore, Singapore; 2 Duke-NUS Medical School, Singapore, Singapore; 3 Department of Emergency Medicine, Tan Tock Seng Hospital, Singapore, Singapore; 4 Accident and Emergency Service, St Mary’s Hospital, London, United Kingdom; 5 Department of Medicine, Imperial College, Kensington, London, United Kingdom; Klinikum rechts der Isar der Technischen Universitat Munchen, GERMANY

## Abstract

**Objectives:**

Febrile infants younger than 3 months old present a diagnostic dilemma to the emergency physician. We aim to describe a large population of febrile infants less than 3 months old presenting to a pediatric emergency department (ED) and to assess the performance of current heart rate guidelines in the prediction of serious infections (SI).

**Materials and methods:**

We performed a retrospective review of febrile infants younger than 3 months old, between March 2015 and Feb 2016, in a large tertiary pediatric ED. We documented the primary outcome of SI for each infant, as well as the clinical findings, vital signs, and Severity Index Score (SIS). We assessed the performance of the Paediatric Canadian Triage and Acuity Scale (PaedCTAS), Advanced Pediatric Life Support (APLS) guidelines and Fleming normal reference values, using sensitivity, specificity, positive predictive value (PPV), negative predictive value (NPV) and area under receiver operating characteristics curve (AUC).

**Results:**

1057 infants were analyzed, with 326 (30.6%) infants diagnosed with SI. High temperature, tachycardia, and low SIS score were significantly associated with SI. Item analysis showed that the SIS performance was driven by the presence of mottling (p = 0.003) and high temperature (p<0.001). The APLS guideline had the highest sensitivity (66.0%, 95% CI 60.5–71.1%), NPV (73.3%, 95% CI 69.7–76.5%) and AUC (0.538), while the PaedCTAS (2 standard deviation from normal) had the highest specificity (98.5%, 95% CI 97.3–99.3%) and PPV (55.2%, 95% CI 32.7–71.0%).

**Conclusions:**

Current guidelines on infantile heart rates have a variable performance. In our study, the APLS heart rate guidelines performed with the highest sensitivity, but no individual guideline predicted for SIs satisfactorily.

## Introduction

Febrile young infants younger than 3 months old present a diagnostic dilemma to the pediatric emergency department (ED) physician. The potential for a missed serious infection (SI) poses the threat of premature death and long-term disability among these infants. [1,2] Despite decreasing early-onset neonatal sepsis rates due to obstetric prevention strategies [3], high rates of hospitalization and administration of parenteral antibiotics occur in this age group. Continual tension remains between the need for early and aggressive intervention among patients suspected with sepsis [4] and the global phenomena of increasing antibiotic resistance. [5,6] Research networks have attempted to build diagnostic algorithms to guide the identification of these ill infants. [7,8] These are often useful as adjuncts to the clinician’s gestalt, but generalizability remains questionable.

Vital signs are of paramount importance in recognizing ill children and have been used in pediatric early warning system scores (PEWS) [9] and various triage systems [10]. Vital signs have resurfaced as the focus of research in recent years, with various groups purposing to update evidence-based normal heart rate ranges among children. [11–16] Normative heart rate ranges are infamously difficult to define due to the hemodynamic lability in these young infants, multiple confounders for abnormal heart rate, and the variable physiological response during acute stress states.

In this study, we aim to (1) describe the presentation and outcomes of a large population of febrile young infants less than 3 months old presenting to a pediatric ED in an Asian city, and (2) assess the performance of the Paediatric Canadian Triage and Acuity Scale (PaedCTAS) [10], the Advanced Pediatric Life Support (APLS) guidelines [11] and the Fleming normal reference values [12], in the prediction of SIs.

## Materials and methods

### Study design and setting

This is a retrospective chart review from 1st March 2015 – 29th Feb 2016 in KK Women’s and Children’s Hospital (KKH). Singapore is a small urban nation of 5.5 million people, with 16% of its population less than 15 years’ old. [17] Among Singapore residents, about 194,432 are under 5 years’ old. [18] KKH is the larger of two pediatric specialty hospitals in the country and patients who attend the ED are charged a nominal fee. The annual ED attendance in KKH is about 174,000 children.

### Participants

We screened all children less than 3 months old with an initial triage axillary temperature of > 37.5°C. Temperature is routinely measured at the triage by a nurse, using an axillary thermometer (Terumo® digital clinical thermometer), for infants < 6 months. Subsequently, if the repeat temperature was less than 38°C (e.g. in cases of overwrapping) and the child remained clinically well with no subsequent investigations performed, this child was excluded from the analysis. Young infants admitted for jaundice or poor feeding but who did not mount a fever response were excluded.

### Variables

#### Triage

Our triage system utilizes the Severity Index Score (SIS) [19] which is interpreted in the following categories: SIS 10 (Not very sick), SIS 8 or 9 (moderately sick), SIS 7 or less (Very sick). The SIS comprises the domains of respiratory effort, color, activity, temperature and play. Heart rate, pulse oximetry and blood pressure (BP) are automated measurements using the Dinamap GE ProCare 300 Vital Signs Monitor, while respiratory rate is manually measured by the triage nurse. BP is measured using the neonatal or infant cuff, as appropriate. On occasions where the neonate appeared well but repeated attempts to obtain the BP failed due to movement artifacts, the triage was based on heart rate and respiratory rate. Our ED triage uses the PaedCTAS guideline for normal heart rate and respiratory rate ranges. [10] For this study, the SIS and vital signs were extracted from the electronic health record.

#### History and physical examination

We included the following perinatal details: gestation and maternal group B streptococcus (GBS) status. Physical examination findings of respiratory distress, lethargy, and capillary refill time were collected.

#### Investigations and management

Biochemical results included total white blood cell count, absolute neutrophil count, hemoglobin, platelets, C-reactive protein, urinalysis, and cerebrospinal fluid (CSF) analysis. Microbiological culture results from urine, blood, CSF, stool and other sites (abscess, wounds), as well as nasopharyngeal aspirate for common viruses were recorded. We documented if the infant received intravenous antibiotics, fluid boluses, inotropes, ventilator support, required intensive care or if they died.

### Primary outcome

SIs were defined as: sepsis (including bacteremia), meningitis, lobar pneumonia (confirmed radiologically), osteomyelitis, abscess, and urinary tract infection. [20]

### Data sources/measurement

Records were reviewed in detail using standardized definitions as above. YWC, MZC, PN and SZO were trained to review the records for the variables listed. Differences were resolved by discussion with SLC. 5% of the data was validated by a second reviewer, YKO, for data accuracy. For PaedCTAS [10], we used both +/-1 SD from normal range (heart rate <90 or >180/min) and +/-2 SD from normal range (heart rate <65 or >205/min) to define abnormal heart rate. For the APLS guidelines [11], we used the single threshold for < 1 year old (heart rate <110 or >160/min). For Fleming et al [12], we used 3-month-old thresholds of <1^st^ or >99^th^ centile (heart rate <107 or >179/min), <5^th^ or >95^th^ centile (heart rate <117 or >168/min), and <10^th^ or >90^th^ centile (heart rate <123 or >162/min).

Ethics approval for this study was granted by the Singapore Singhealth Institutional Review Board, with waiver of informed consent.

### Statistical methods

Categorical data were summarized by frequencies (and percentages) and continuous data by means (and SD) or medians (and interquartile ranges (IQRs)). Where data approached normality, we presented the data with means (and SDs) based on the central limit theorem. Fisher’s exact test or chi square test was performed to assess for association between SI and categorical predictors of interest, while t-test or Wilcoxon rank sum was used for continuous variables, depending on normality of data. We presented the performance of the PaedCTAS [10], APLS guidelines [11] and Fleming normal reference values [12] using sensitivity, specificity, positive predictive value (PPV) and negative predictive value (NPV). We also described the performance of heart rate and each guideline using the area under receiver operating characteristics (ROC) curve (AUC). Significance was taken at a p value of less than 0.05. The analysis was performed using IBM SPSS Statistics v23.0.

## Results

**Fig 1:**
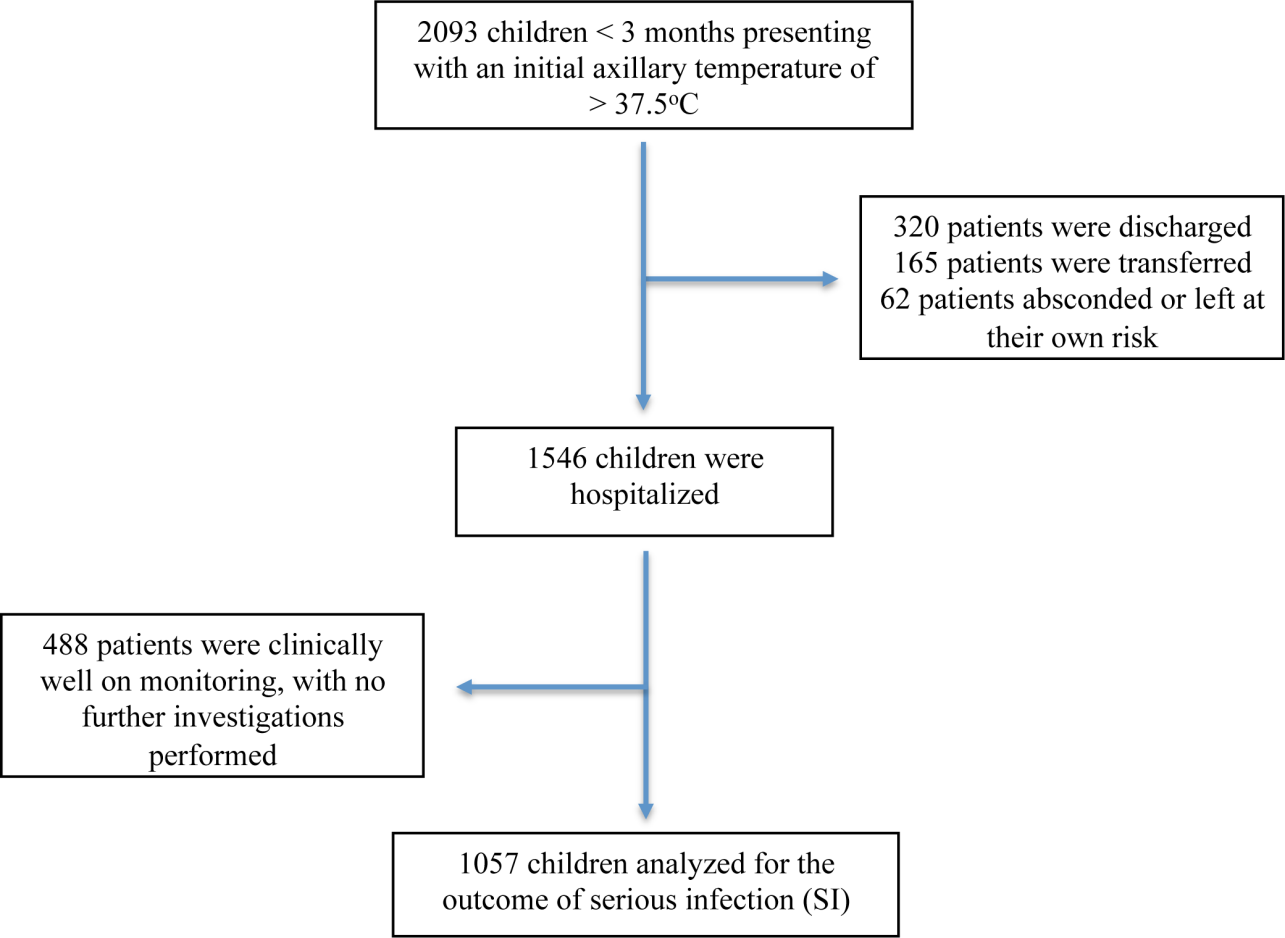
Flow diagram of study population.

Out of 2093 young infants < 3 months who presented with an initial axillary temperature of > 37.5°C, 99 infants (4.7%) were triaged as most urgent and were required to be seen immediately, while 1161 (55.5%) were triaged as urgent and required to be seen within 15 minutes of triage. Vital signs, triage status and ED disposition are described in Table 1. Among the 2093 infants, 1546 were hospitalized and 1057 were analyzed for the outcome of SIs. (Fig 1) Of these, 247 infants (23.4%) were younger than 1 month old and 326 infants (30.8%) were diagnosed with SI. (S1 Dataset)

**Table 1:** Demographics and initial presentation of all infants (n = 2093).

Age in months, mean (SD)	1.1 (1.0)
Ethnicity, n (%)	
Chinese	1170 (55.9)
Malay	496 (23.7)
Indian	209 (10.0)
Others	218 (10.4)
Temperature in ^o^C, mean (SD)	38.0 (0.5)
Heart rate per minute, mean (SD)	159 (21)
Respiratory Rate per minute, mean (SD)	42 (7)
Pulse oximetry %, mean (SD)	98.6 (1.8)
Triage category^a^, n (%)	
Priority 1	99 (4.7)
Priority 2A	1161 (55.5)
Priority 2	829 (39.6)
Priority 3	4 (0.2)
Normal saline bolus given, n (%)	95 (4.5)
Paracetamol served, n (%)	54 (2.6)
Disposition, n (%)	
Admitted to intensive care unit or acute care ward	39 (4.5)
Admitted to general ward	1507 (72.0)
Discharged	320 (15.3)
Discharged against advice or absconded	62 (3.0)
Transferred or others	165 (7.9)

^a^Triage category reflects the mandated time to be seen after completion of triage: Priority 1 –seen immediately; Priority 2A –seen within 15 mins; Priority 2 –Seen within 1 hour; Priority 3 –Seen within 4 hours

In the univariate analysis (Table 2), the triage parameters of temperature (p<0.001), heart rate (p = 0.006), SIS score category (p = 0.005) and triage category (p = 0.019) had a statistically significant relationship with the outcome of SI. Item analysis showed that the SIS performance was driven by color interpreted as the presence of mottling (p = 0.003), and the temperature of the child (p<0.001). (Table 3)

**Table 2:** Univariate analysis of predictors for serious infections, for hospitalized patients (n = 1057).

	No Serious Infection (n = 731)	Serious Infection (n = 326)	p value^a^
Age in months, mean (SD)	1.4 (1.0)	1.4 (0.9)	0.419
Male Gender, n (%)	394 (53.9)	235 (72.1)	<0.001
Temperature in ^o^C, mean (SD)	38.3 (0.5)	38.5 (0.6)	<0.001
Heart Rate per min, mean (SD)	165 (21)	169 (20)	0.006
Respiratory Rate per min, mean (SD)	42 (6)	42 (7)	0.630
SpO2 (%), mean (SD)	98.6 (2.5)	98.7 (1.7)	0.438
SIS^b^ score in categories, n (%)			0.005
10	221 (30.2)	68 (20.9)	
9 or 8	486 (66.5)	243 (74.5)	
≤ 7	24 (3.3)	15 (4.6)	
Triage category^c^, n (%)			0.019
Priority 1	52 (7.1)	31 (9.5)	
Priority 2A	116 (15.9)	32 (9.8)	
Priority 2	563 (77.0)	263 (80.7)	
Respiratory distress/retractions, n (%)	13 (1.8)	9 (2.8)	0.351
Toxic in appearance, n (%)	14 (1.9)	10 (3.1)	0.266
Capillary refill time > 2 secs, n (%)	24 (3.3)	16 (4.9)	0.222
Day of illness, mean (SD)	2.3 (2.6)	2.0 (2.3)	0.126
Prematurity, n (%)	63 (8.6)	27 (8.3)	0.905
Pre-exiting comorbidities, n (%)	28 (3.8)	14 (4.3)	0.734
Maternal GBS^d^, n (%)			0.603
Present	142 (19.4)	55 (16.9)	
Absent	365 (49.9)	166 (50.9)	
Unknown	224 (30.6)	105 (32.2)	
Fluid bolus in the ED, n (%)	50 (6.8)	35 (10.7)	0.037
Investigations			
Total white blood cell count, mean (SD)	12.1 (5.3)	14.0 (6.5)	<0.001
Absolute neutrophil count, mean (SD)	5.3 (4.2)	6.8 (4.4)	<0.001
Platelets, mean (SD)	414 (128)	446 (149)	0.001
C-reactive protein, mean (SD)	12.7 (23.1)	34.8 (50.1)	<0.001
Post Admission			
Fluid bolus, n (%)	61 (8.3)	39 (12.0)	0.069
Inotropes, n (%)	3 (0.4)	3 (0.9)	0.380
Intravenous ampicillin, n (%)	484 (66.2)	305 (93.6)	<0.001
Intravenous gentamicin, n (%)	477 (65.3)	308 (94.5)	<0.001
Ventilatory support, n (%)	9 (1.2)	7 (2.1)	0.280
Intensive care unit stay, n (%)	7 (1.0)	7 (2.1)	0.145
Duration of hospitalization, mean (SD)	3.1 (2.5)	4.5 (4.2)	<0.001

^a^p value from Fisher’s exact test for categorical variables and t-test for continuous variables

^b^SIS–Severity Index Score

^c^Triage category reflects the mandated time to be seen after completion of triage: Priority 1 –seen immediately; Priority 2A –seen within 15 mins; Priority 2 –Seen within 1 hour; Priority 3 –Seen within 4 hours

^d^Group B Streptococcus

**Table 3:** Item analysis of Severity Index Score (SIS).

	No Serious Bacterial Infection (n = 731)	Serious Bacterial Infection (n = 326)	p value^a^
Respiratory Distress	42 (5.7)	13 (4.0)	0.294
Activity			0.210
Stupor or Coma	0	1 (0.3)	
Lethargy	10 (1.4)	7 (2.1)	
Normal	721 (98.6)	318 (97.5)	
Mottled color	41 (5.6)	36 (11.0)	0.003
Play			0.323
Refused	3 (0.4)	1 (0.3)	
Decreased	327 (44.7)	162 (49.7)	
Normal	401 (54.9)	163 (50.0)	
Temperature			< 0.001
<36.3 and > 40.0	2 (0.3)	2 (0.6)	
38.4–40.0	274 (37.5)	171 (52.5)	
36.3–38.3	455 (62.2)	153 (46.9)	

^a^p value from Fisher’s exact test

Investigations done showed that the total white blood cell count, absolute neutrophil count, platelets, and C-reactive protein were statistically significant in predicting for SI in our population. Only 19 (5.8%) infants with SI had a positive nasopharyngeal aspirate for common viruses, compared to 139 (19.0%) infants with no SI (p<0.001), with an overall positivity rate of 14.9%. A significantly larger proportion of infants with SI received intravenous antibiotics and had a longer duration of hospitalization, compared to infants without SI. (Table 2)

Among the SIs, urinary tract infections were the most common, with E Coli and Klebsiella as the most likely pathogens. (Table 4) This was followed by meningitis and septicemia. The breakdown between viral and bacterial causes of meningitis (Table 5) showed that there was no statistically significant difference in vital signs or blood investigation results.

**Table 4:** Serious infections (SI) by body system.

Type of SI	Urinary Tract Infection (n = 178)	Meningitis (n = 112)	Septicemia (n = 21)	Lobar Pneumonia (n = 7)	Others^a^ (n = 8)
Male gender, n (%)	142 (80)	70 (63)	16 (76)	4 (57)	3 (38)
Heart rate, mean (SD)	168 (23)	170 (15)	165 (17)	171 (20)	171 (12)
Respiratory rate, mean (SD)	42 (7)	43 (6)	40 (6)	47 (12)	42 (4)
Temperature, mean (SD)	38.5 (0.5)	38.5 (0.5)	38.4 (0.7)	38.3 (1.2)	38.2 (0.8)
SIS, mean (SD)	8.8 (0.9)	8.8 (0.8)	8.6 (1.1)	8.1 (2.1)	9.5 (0.5)
Total white cell count, mean (SD)	15.9 (6.8)	10.7 (4.2)	15.1 (7.0)	13.3 (5.4)	19.0 (8.2)
Absolute neutrophil count, mean (SD)	7.8 (4.5)	4.4 (2.8)	9.2 (5.3)	6.8 (4.9)	8.9 (7.1)
Platelets, mean (SD)	459 (150)	432 (134)	360 (137)	452 (187)	582 (204)
C-reactive protein, mean (SD)	41.4 (46.7)	11.7 (25.6)	85.4 (77.4)	53.3 (88.4)	60.3 (91.4)
Most common organisms	E Coli Klebsiella pneumoniae Enterococcus faecalis	Enterovirus E Coli	Group B Streptococcus E Coli Klebsiella pneumoniae		

^a^Others: Enterocolitis, abscess, omphalitis, or cellulitis

**Table 5:** Vital signs and biochemical investigations in viral and bacterial meningitis.

Type of Meningitis	Viral Meningitis (n = 91)	Bacterial Meningitis (n = 21)	p value
Heart Rate, mean (SD)	170 (14)	171 (18)	0.709
Respiratory Rate, mean (SD)	43 (6)	42 (7)	0.692
Temperature, mean (SD)	38.5 (0.5)	38.5 (0.5)	0.838
SIS, mean (SD)	8.8 (0.7)	8.8 (0.9)	0.782
Total white cell count, mean (SD)	10.5 (4.0)	11.6 (5.0)	0.265
Absolute Neutrophil Count, mean (SD)	4.3 (2.5)	5.1 (3.6)	0.229
Platelets, mean (SD)	424 (118)	465 (188)	0.357
C-reactive Protein, mean (SD)	8.6 (12.5)	24.6 (51.8)	0.175
Most common organism	Enterovirus	E Coli	

Table 6 shows the performance of the PaedCTAS [10], APLS guidelines [11] and the Fleming normal reference values [12] in our study population. The APLS and Fleming (<10^th^ or >90^th^ centile) performed with the highest sensitivity (66.0% and 62.6%, respectively) and the highest NPV (73.3% and 71.4%, respectively). No single guideline reached a sensitivity of greater than 70%. Guidelines with more stringent criteria that define tachycardia (PaedCTAS with 1 or 2SD from normal range, and Fleming {<1^st^ or >99^th^ centile) performed with better specificity (78.1%, 98.5% and 73.9%, respectively). The AUC for heart rate was 0.548. The AUC for each guideline was: 0.506 (PaedCTAS 1SD from normal range), 0.511 (PaedCTAS 2SD from normal range), 0.538 (APLS), 0.522 (Fleming <10^th^ or >90^th^ centile), 0.525 (Fleming < 5^th^ or >95^th^ centile), 0.506 (Fleming < 1^st^ or >99^th^ centile).

**Table 6:** Performance of current pediatric heart rate guidelines.

	Total No. with abnormal HR (n = 1057)	Sensitivity (%) (95% CI)	Specificity (%) (95% CI)	PPV (%) (95% CI)	NPV (%) (95% CI)
PaedCTAS^a^ [10] HR^b^ < 90 or > 180 per min (1 SD from normal range)	235 (22.2)	23.0 (18.6–28.0)	78.1 (74.9–81.1)	31.9 (26.9–37.4)	69.5 (68.0–70.9)
PaedCTAS^a^ [10] HR^b^ < 65 or > 205 per min (2 SDs from normal range)	23 (2.2)	3.7 (1.9–6.3)	98.5 (97.3–99.3)	52.2 (32.7–71.0)	69.6 (69.1–70.1)
APLS^c^ [11] HR^b^ < 110 or > 160 per min	642 (60.7)	66.0 (60.5–71.1)	41.6 (38.0–45.3)	33.5 (31.3–35.7)	73.3 (69.7–76.5)
Fleming et al [12] HR^b^ <123 or >162 per min (<10^th^ or >90^th^ centile)	630 (59.6)	62.6 (57.1–67.9)	41.7 (38.1–45.4)	32.4 (30.2–34.7)	71.4 (68.0–74.7)
Fleming et al [12] HR^b^ <117 or >168/min (<5^th^ or >95^th^ centile)	515 (48.7)	52.1 (46.6–57.7)	52.8 (49.1–56.5)	33.0 (30.2–35.9)	71.2 (68.4–73.9)
Fleming et al [12] HR^b^ <107 or >179/min (<1^st^ or >99^th^ centile)	280 (26.5)	27.3 (22.5–32.5)	73.9 (70.5–77.0)	31.8 (27.3–36.6)	69.5 (67.8–71.1)

^a^PaedCTAS–Paediatric Canadian Triage and Acuity Scale

^b^HR–Heart Rate

^c^APLS–Advanced Pediatric Life Support

## Discussion

In this study, we described the triage, diagnostic parameters and clinical outcomes of a large Asian cohort younger than 3 months’ old presenting to a pediatric ED with fever. We demonstrated that a low SIS, together with abnormal heart rate and high temperature, significantly predicted for SI. We also showed the performance of current published heart rate normal ranges in our population.

Despite heart rate being a universally recognized triage tool, heart rate normal ranges vary between guidelines [10–12] and remain difficult to validate in different pediatric populations. In a healthy cohort of Chinese children in Hong Kong, investigators found that a significant number of children had vital signs that fell outside the APLS age-based reference ranges. [21] In our study on infants younger than 3 months old, when compared to other heart rate guidelines and reference values, the APLS [11] guideline appeared to perform with the highest sensitivity and AUC. However, none of the guidelines reached satisfactory performance to be used singly as a predictive tool.

There was significant overlap in the heart rate distributions between infants with and without SIs, suggesting that heart rate alone is unlikely be discriminatory between the two groups. This was highlighted by the AUC of 0.548 for heart rate. We do recognize that single read-outs of a child’s heart rate are easily confounded and may be inferior to an analysis of heart rate variability.[17] Research in neonatal intensive care units report that heart rate characteristic monitoring is useful in reducing mortality rate among very low birth weight infants. [22,23] These results have not been demonstrated in the ED setting, where heart rate variability may have an increasing role to play in assisting the ED physician’s assessment of ill infants.

Clinical decision-making is often complemented by judicious use of biomarkers, including white blood cell count, absolute neutrophil count, C-reactive protein and interleukin-6. [24,25] More recent work has focused on the association of RNA biosignatures with bacterial infections in young infants 60 days or younger. [26] In our study, we showed that a high total white blood cell count, absolute neutrophil count and C-reactive protein significantly predicted for SIs. We do not perform tests on interleukins or RNA markers among febrile infants in our institution.

In this study, we addressed the important question of how current heart rate guidelines perform when predicting which febrile young infant has a serious infection. We highlight that despite robust attempts at quantifying normal heart rate ranges [10–12], the utility of these ranges in providing guidance to ED physicians who manage febrile young infants at risk of serious infections remains limited. ED physicians continue to rely on a constellation of vital signs, symptoms and signs to make clinical decisions, while awaiting a vital signs tool that can provide greater discriminatory power.

We recognize the limitations in our study. Firstly, being a retrospective study, the reviewers of health records were at risk for misclassification bias and were not blinded to the aims of the study. This however, would not have affected objective measurements like heart rate, temperature, or investigation results. Secondly, respiratory rate was manually measured at triage. Inaccuracies in this single measurement could have resulted in the non-significant association between respiratory rate and SIs in our study population. Thirdly, in choosing the primary outcomes of SIs, we included viral meningitis because of concerns of the long-term sequelae of such infections. We did demonstrate that there was no statistically significant difference in vital signs or blood investigation results between the viral and bacterial meningitis groups in our study population. Fourthly, in our study, we examined original heart rates without adjustment. This was to reflect the need for rapid dichotomous decisions at the ED setting in this young population. Finally, this is a single center study and our findings require validation in a new population.

In conclusion, we described a large population of febrile infants younger than 3 months’ old presenting to the ED. In our population, the APLS heart rate guidelines performed with the highest sensitivity, but no individual guideline predicted for SIs satisfactorily.

## Supporting information

S1 Dataset Data file for febrile infants. (XLSX)
